# A Modified Effect on Asthma: Ozone and Secondhand Smoke Outweigh Genetic Influence

**Published:** 2007-04

**Authors:** Carol Potera

Individual variations in genes, known as single-nucleotide polymorphisms (SNPs), help to explain why some children are more susceptible to asthma and allergies. But does exposure to ozone or secondhand smoke alter this genetic susceptibility? Public health experts from Mexico and the United States report that, in 596 families with asthmatic children living in Mexico City, where ozone levels rank as the highest in North America, parental smoking can indeed modify the risk conferred by a particular SNP **[*EHP* 115:616–622; Wu et al.]**.

Complex interactions among genes and environmental triggers are known to contribute to asthma and allergic reactions in children. Exposure to ozone, for instance, turns on the *TNF* gene for the production of tumor necrosis factor-α, a cytokine that causes airway inflammation. So does exposure to cigarette smoke.

The children, who ranged in age from 4 to 17 years, largely had mild asthma. Half lived with a smoking parent. The researchers measured variations in *TNF* and the gene for lymphotoxin-α (*LTA*) in the children and their parents. *TNF* and *LTA* lie next to each other on chromosome 6 and share receptors. Two SNPs for *LTA* and four for *TNF* capture most of the variation in these two genes.

The team found that *LTA* was not associated with asthma risk, but one SNP for *TNF* (coded 308A) raised the risk by 50% among all children. This SNP and one other (238A) more than doubled the risk of asthma among children living with nonsmoking parents. None of the SNPs for *LTA* or *TNF* were linked to asthma among children living with smoking parents. In addition, allergic reactions generated with skin prick tests were not related to any of the SNPs tested.

The researchers suspect that secondhand smoke and ozone may synergistically increase production of tumor necrosis factor-α, an effect that overrides the minor influence of genetic variation. Therefore, the effects of certain SNPs on risk of asthma may stand out more clearly in children who are not exposed to secondhand smoke.

## Figures and Tables

**Figure:**
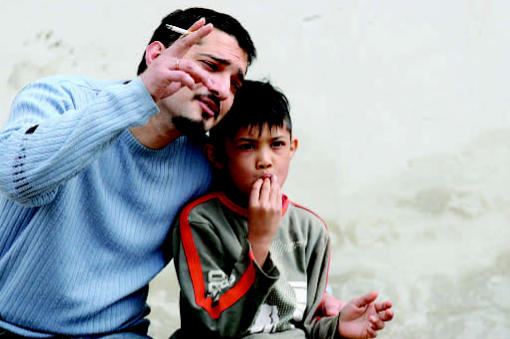
Smoke screen? For certain people, secondhand smoke exposure may override genetic influence on asthma risk.

